# Body Pigmentation as a Risk Factor for the Formation of Intracranial Aneurysms

**DOI:** 10.1155/2014/301631

**Published:** 2014-05-22

**Authors:** Günter Schulter, Klaus Leber, Elke Kronawetter, Viktoria R. Rübenbauer, Peter Konstantiniuk, Ilona Papousek

**Affiliations:** ^1^Department of Psychology, Biological Psychology Unit, Karl-Franzens-University, Universitätsplatz 2/III, 8010 Graz, Austria; ^2^Department of Neurosurgery, Medical University, Auenbruggerplatz 2, 8036 Graz, Austria; ^3^Department of Surgery, Division of Vascular Surgery, Medical University, Auenbruggerplatz 2, 8036 Graz, Austria

## Abstract

Recent studies demonstrated pigmented cells both in the murine heart, in pulmonary veins, and in brain arteries. Moreover, a role for melanocytes in the downregulation of inflammatory processes was suggested. As there is increasing evidence that inflammation is contributing significantly to the pathogenesis of intracranial aneurysms, melanocyte-like cells may be relevant in preventing age-related impairment of vessels. As pigmentation of the heart reflects that of coat color, aspects of body pigmentation might be associated with the incidence of intracranial aneurysms. We performed a case-control study to evaluate associations between the pigmentation of hair and eyes and the formation of aneurysms. In addition to hair and eye color, constitutive and facultative skin pigmentation were assessed in a replication study as well as individual handedness which can be seen as a neurophysiological correlate of developmental pigmentation processes. Hair pigmentation was highly associated with intracranial aneurysms in both samples, whereas eye pigmentation was not. In the replication cohort, facultative but not constitutive skin pigmentation proved significant. The strongest association was observed for individual handedness. Results indicate a significant association of intracranial aneurysms with particular aspects of body pigmentation as well as handedness, and imply clinical usefulness for screening of aneurysms and possible interventions.

## 1. Introduction

The pathogenesis of intracranial aneurysm formation and rupture remains mostly unknown. Current evidence favors an interaction of multiple environmental [1, 2] and genetic factors [3, 4] in the initiation and progression of intracranial aneurysms. However, there is a growing body of evidence that inflammation is contributing significantly to the pathogenesis of intracranial aneurysms. Starting with an endothelial dysfunction, an increasing inflammatory response results in a modulation of vascular smooth muscle cells and, finally, in an apoptotic degradation of the vascular wall giving rise to aneurysmal dilation and progression [5–7].

Nevertheless, the various results on the involvement of inflammation in aneurysm formation raise another still unresolved issue, in particular why only in some individuals several unfavorable conditions (e.g., smoking, oxidative and hemodynamic stress, and others) result in serious inflammatory responses and, consequently, in aneurysm formation, but in the vast majority of individuals they do not. Therefore, any new perspective in aneurysm research may help to increase the understanding of etiological processes and, consequently, may improve screening methods and therapeutic interventions. One such new and possibly fruitful approach relates to recent studies investigating the distribution and function of “non-classical” melanocytes, that is, pigmented cells which are not located in the skin and are not involved in skin pigmentation. Such nonclassical melanocytes are located in sites as different as the eye, inner ear, bones, and meninges. Additionally, recent evidence exists that there is also a consistent distribution of pigmented cells in several structures of the mouse heart [8, 9]. Moreover, there is also evidence for the existence of melanocyte-like cells in murine and human vessels, that is, cells populating the endothelium of pulmonary veins, expressing both the melanin synthesis enzyme dopachrome tautomerase (Dct) and melanoblast markers, and some of them even containing mature melanosomes [10]. Finally, and most importantly for the present study, the presence of melanocytes was also verified in murine brain arteries, with a particularly high concentration of melanocytes observed in the wall of middle cerebral arteries and in several of their branches on the basal brain surface. The melanocytes were mainly multipolar and often formed a plexus around blood vessels [11].

Functionally, such “non-classical” melanocytes in the heart, for instance, may be involved in atrioventricular valve development, but a recent study also demonstrated that the mechanical properties along the leaflet vary with the degree of pigmentation; that is, the viscoelastic properties of the valves and their proper functioning were affected by the presence of melanocytes [12]. Furthermore, a Dct-dependent intermediate of the melanin synthesis was already established as a potent hydroxyl radical-scavenging agent [13], indicating a possible role for melanocytes in murine and human vessels in the regulation of antioxidation processes, too. As long-lasting (mitochondrial) oxidative stress is causing deleterious changes in vascular walls [14], Dct-expressing cells, therefore, may also be relevant in preventing age-related impairment of vessels such as the formation of aneurysms. Of particular importance, however, are various anti-inflammatory and immunomodulatory properties of the melanocortin system. A lot of recent studies have demonstrated potent anti-inflammatory effects of alpha-melanocyte-stimulating hormone (*α*-MSH) mediated by melanocortin receptors of different subtypes (MC1R to MC5R). Although *α*-MSH/MC1R interactions primarily contribute to the regulation of skin physiology, MC1 receptor functions extend well beyond the regulation of melanogenesis as it is expressed in a variety of cells, especially in cells of the immune system, that is, macrophage/monocytic cells, lymphocytes with antigen-presenting and cytotoxic functions, neutrophils, and also peripheral blood-derived dendritic cells, downregulating the production of specific proinflammatory cytokines [15–17]. Moreover, and most important for the present study, MC1 receptors are also expressed in endothelial cells [18–20].

Therefore, individual differences in the melanocortin system (determined, inter alia, by single nucleotide polymorphisms in the MC1R gene) should be correlated with individual differences in the regulation of inflammatory processes and, consequently, with a different disposition towards the formation of intracranial aneurysms. As the individual level of pigmentation in the heart is correlated with the individual coat color in animals [21], we assumed that individual differences in the quantity and/or functionality of neural crest-derived pigment cells in hair follicles and iris may mirror individual differences in the number and/or functioning of neural crest-derived nonclassical melanocytes and/or in the functioning of melanocortin 1 receptors in vascular and cardiovascular tissues. Moreover, pericytes and smooth muscle cells of the ventral/anterior compartment of blood vessels in the brain (including the anterior part of the Circle of Willis) have also been shown to originate exclusively from such (cephalic) neural crest progenitor cells [22]. As in neural crest lineage segregation melanocytic and myofibroblastic precursors develop from common intermediate pluripotent neural crest cells and only then differentiate to either melanocytes or pericytes and smooth muscle cells, respectively [23], a significant association between individual hair and eye pigmentation and the disposition and functionality of intracranial vasculature was hypothesized. Moreover, as a majority of intracranial aneurysms are localized at the anterior part of the Circle of Willis [24], empirical evidence for such an association may be of special importance for etiological models of intracranial aneurysm formation.

## 2. Materials and Methods

The study was designed as a case-control study. Patients with a diagnosed intracranial aneurysm were compared with healthy controls with respect to degree of pigmentation of hair and eyes.

### 2.1. Study Population

We collected data in a sample of 53 consecutive adult patients of the same ethnic group (white Europeans) within a period of six months. The only selection criterion was at least one diagnosed intracranial aneurysm; that is, there were no further restrictions regarding, for instance, age, sex, smoking status, or rupture. There was an incidence of rupture/subarachnoid hemorrhage in 29 out of 53 cases. Patients were met at their follow-up check at the outpatient clinic of the Department of Neurosurgery of the Medical University of Graz during the time of waiting for their medical examination, about six months after they had undergone successful treatment. A total of 136 adult controls were recruited by advertisements in a local newspaper, with reference to an ongoing study on human pigmentation in a quite different setting (i.e., in a study at the University of Natural Sciences), so that patients and controls were very probably unrelated although both came from the same local area. An even distribution of men and women was realized. Controls were also white Europeans, were paid for their participation, and were without any currently existing health problems or long-term medication according to their own statements and with no known intracranial aneurysm. Assuming an average prevalence of 2-3% for an asymptomatic intracranial aneurysm [25], three or four individuals out of 136 controls may have had an incidental cerebral aneurysm, which is statistically neglectable. Moreover, any assimilation of controls to patients will increase the odds against detecting statistically significant differences. Detailed sample characteristics are given in Table 1.

Written informed consent was obtained from all participants, and the study protocol was approved by the Institutional Review Board of the Medical University of Graz. After recording participants' date of birth and smoking status (smoker or ex-smoker versus nonsmoker), hair and eye pigmentation was assessed.

### 2.2. Hair Color

Pigmentation levels were objectively determined by comparing patient's hair with seven natural hair samples selected from standard hair color swatches, a collection routinely used in physical anthropology and medicine (Fischer and Saller color standards; DKSH, Zürich, Switzerland). Pigmentation was classified on a numeric scale from 1 (strong pigmentation, that is, dark brown) to 7 (weak pigmentation, that is, strawberry blonde/red-haired; red hair contains greater contents of pheomelanin relative to eumelanin, but both pigments are on a low level) [26]. In four/two patients/controls with greyed hair and in two/five patients/controls with dyed hair, either inspection was done close to the scalp or—if whitening/coloring was comprising the whole hair shaft—original hair color was estimated by the color of the eyebrows and by the participant him-/herself. To further optimize reliability of ratings and to facilitate a straightforward interpretation of results, the seven grading values were pooled into three global color groups: dark brown to brown, light brown to dark blond, and blond to light blond or strawberry blonde/red-haired.

### 2.3. Eye Color

The patient's eye color was objectively determined by comparing it with ten images of natural eyes representing the whole spectrum of eye colors. Eye pigmentation was classified on a numeric scale from 1 (strong pigmentation, that is, dark brown) to 10 (weak pigmentation, that is, light blue). Ratings were always done under identical light conditions. To optimize reliability, the ten grading values were also pooled into three global eye color groups: dark color (shades of brown), mixed color (blue or green with admixture of brown pigment), and light color (shades of blue or gray).

### 2.4. Statistical Analysis

Multivariate logistic regression was used to estimate odds ratios in predicting the occurrence of intracranial aneurysms. Owing to the collinearity of the different facets of pigmentation, the effect of each pigmentation variable on the risk of aneurysm formation was evaluated in separate multivariate models. Each model was adjusted for age (treated as a continuous variable), smoking, and sex to correct for possible effects of these variables on pigmentation. Odds ratios were calculated for particular subgroups of pigmentation to facilitate interpretation of odds ratios associated with a respective subtype. Moreover, to evaluate trends in odds, ordinal variables were modeled as single, continuous, and independent variables. Additionally, an estimation (by Nagelkerke *R* ^2^ coefficient) of the percentage of variation explained by the developed model is presented, but we only reported the amount of explained variance which is added by the relevant pigmentation variable (or by handedness) in our stepwise logistic regression model, excluding the contributions by the covariates (see last column: *R* ^2^+ in Tables 2, 3, and 4). Analyses were done using SPSS 18 (IBM Corporation, Somers, NY, USA).

## 3. Results

Multivariate logistic regression was done on the three global groups of hair and eye color, respectively. Respective analyses showed different results for hair and eye pigmentation, with odds ratios adjusted for age, smoking, and sex (Table 2). Hair color showed a pronounced effect in predicting the occurrence of intracranial aneurysms, indicating an increasing degree of association with decreasing pigmentation of hair (*P* for trend <0.001). Compared to dark colored hair, blond hair and very light hair were highly associated with intracranial aneurysm (odds ratio: 23.50; 95% confidence interval [CI]: 3.99 to 138.24). By contrast, the regression with pigmentation levels of eyes as explanatory variable showed no significant effect (Table 2).

## 4. Replication Study: Materials and Methods

In view of these inconsistent results, we conducted a replication study, because replication is strongly recommended by statisticians as the best validation strategy [27]. For this purpose, another independent sample of patients with diagnosed intracranial aneurysms was tested. The same methods and statistical analyses were applied in both samples. However, the control group of Sample II differed from Sample I in so far as in all participants it was angiographically ensured that there was no incidence of an intracranial aneurysm. Furthermore, in addition to the assessment of hair and eye color, constitutive and facultative skin pigmentation were determined. Moreover, for the common variance between brain lateralization and body pigmentation (both presumably mirroring characteristics and common pathways in the development of the cephalic neural crest), handedness was assessed.

Body pigmentation is associated with specific functional characteristics of the central nervous system. In the visual system, for instance, a misrouting of the optic nerves in human albinism, that is, an abnormal projection of the temporal retina to the contralateral hemisphere, is well documented [28]. Moreover, there is evidence for a significant relationship between the extent of such deviant functional brain lateralization and the individual degree of hair and eye pigmentation [29]. Similar associations have been shown for the albino auditory system [30] and also for the motor system in humans without distinct features of albinism [31]. A higher frequency of non-right-handedness, that is, an anomalous motor lateralization, was observed in subjects with light pigmentation of their hair.

### 4.1. Study Population

Half a year after finishing data collection for Study I, we started data collection in an independent sample of 58 consecutive white European patients, also within a period of six months and following the same procedure and selection criteria as in Study I. There was an incidence of rupture/subarachnoid hemorrhage in 34 out of 58 cases. Deviating from Study I, however, controls were recruited at the Department of Surgery, Division of Vascular Surgery of the Medical University of Graz. They were 38 white European patients who were medically examined and treated for carotid stenosis and, therefore, possible intracranial aneurysms were reliably excluded by the use of intracranial angiography. Control patients were tested during their in-patient stay at the second postoperative day after having given written informed consent. The protocol was approved by the Institutional Review Board of the Medical University of Graz. Detailed sample characteristics are given in Table 1.

Date of birth, smoking status, and hair and eye pigmentation were determined using the same methods as in Study I. Additionally, constitutive and facultative skin pigmentation were assessed as well as direction and extent of handedness.

### 4.2. Skin Color

To objectively determine constitutive skin pigmentation [32], the surface of a scanner (CanoScan Lide 90, Colour Image Scanner) was covered by a sheet of paper with a squared area of 5 × 7 cm left open. The inner surface of the left and right upper arm was scanned about 5 cm proximally to the crook of the arm. The resulting image was standardized to a size of 800 × 570 pixels with a 24-bit color resolution (using Microsoft Picture Manager). To each of the three color components “Red,” “Green,” and “Blue,” intensity values (RGB values, ranging from 0 = no color to 255 = bright color) were then assigned (using Adobe Photoshop software). Averaging the RGB values of all pixels and both arms resulted in a basic score of skin pigmentation. Statistical analysis was then based on terciles of these mean scores, defining subgroups of dark, medium, and light skin pigmentation.

### 4.3. Skin Phototype

Whereas constitutive skin color designates a genetically determined level of cutaneous melanin, facultative pigmentation designates the ability to adjust melanization of epidermal cells after sunlight exposure. The “Physician-diagnosed Skin Photo-Type” system (PSPT) [33] was used, asking for the participants' skin responses after a first average sun exposure. Subjects were categorized into three subgroups of photosensitivity: rarely/never burns, tans profusely (PSPT V + VI), burns moderately/minimally, tans moderately (PSPT III + IV), and burns easily, tans never/minimally (PSPT I + II).

### 4.4. Handedness

Handedness was assessed by an objective and standardized paper and pencil test [34] consisting of tasks which had to be performed with the left and the right hand as quickly as possible. A laterality coefficient [(*L* − *R*)/(*L* + *R*)∗100] was calculated as a measure of degree of handedness, with positive values indicating left-handedness. Statistical analysis was based on terciles of laterality coefficients, defining subgroups of pronounced right-handed, moderate right-handed, and bimanual or left-handed subjects.

## 5. Replication Study: Results

The same statistical methods were applied as in Study I. One aneurysm patient with a handedness score of *z* > 3.5 (unrealistically extreme left-handedness) was excluded from the respective analysis, reducing the number of cases to 57. Multivariate logistic regression was done on the three global groups of hair and eye color, as well as on the three subgroups of photosensitivity. Results of case-control comparisons are shown in Table 3. The analysis of hair color yielded virtually identical results as in Sample I (*P* for trend = 0.001). Compared to dark pigmented hair, light pigmentation was highly associated with intracranial aneurysm (odds ratio: 29.11; 95% CI: 3.78 to 224.32). While positive findings for hair color were fully replicated, associations between eye pigmentation and aneurysms were again nonsignificant.

Skin phototype as an explanatory variable proved significant, while skin color did not. The odds ratio for subjects reporting easy burning and minimal/no tanning was 6.08 (95% CI: 1.11 to 33.45) in comparison to rarely/never burning and profusely tanning participants, with a *P* for trend of 0.04. The strongest association was observed for individual handedness. Compared to subjects with pronounced right-handedness, the odds of an intracranial aneurysm were 22.36 (95% CI: 2.64 to 189.32) for subjects with moderate right-handedness and increased to 64.09 (95% CI: 5.40 to 760.69) for bimanual or left-handed subjects. The degree of association increased with an increasing shift from right- to left-handedness (*P* for trend = 0.001).

To give a rough estimate of their predictive power as a screening tool, a logistic regression was additionally calculated with a combination of those independent variables in the data set of Study II which had revealed significant associations with case-control status, that is, hair color, skin photosensitivity, and handedness. For this purpose, the respective global group values for each variable were averaged for each individual and modeled as single, continuous, and independent variable and case-control status was modeled as the dependent variable. Logistic regression showed a highly significant result (Wald statistic = 12.3, df = 1, and *P* < 0.000), with the following classification parameters: sensitivity = 94.7% and specificity = 86.8%; that is, 94.7% and 86.8% of patients and controls, respectively, were classified correctly. Accordingly, the false positive/negative rates were 13.2 and 5.3 percent.

Finally, we combined the data from both samples and calculated two logistic regressions to get a more reliable estimation of odds ratios and, especially, their confidence limits. A combination was possible for the variables of hair and eye color determined in both samples, resulting in a data set of 111 patients and 174 controls. Results of these regression analyses are given in Table 4 and reveal remarkable facts in comparison to the results reported in Tables 2 and 3. Firstly, even the odds ratio for the particular state “light brown to dark blond hair” reaches significance in comparison with “dark brown to brown hair”; that is, even a modest reduction in dye intensity is associated with a small but significant increase in the risk of aneurysm formation: the most conservative estimation of the particular odds ratio, that is, the lower confidence limit, is still 1.45 for individuals with light brown to dark blond hair compared to those with dark brown or brown hair. This is also supported by the significant trend observed already in separate analyses of Samples I and II (*P* < 0.001). Secondly, the odds ratio for the particular state “blond to strawberry blonde/red hair” and the respective confidence limits are substantially reduced indicating a lower error of measurement. Moreover, the lower confidence limit still amounts to about 4, that is, a remarkable value which is based on both data sets and therefore a more valid parameter.

## 6. Discussion

Intracranial aneurysms were clearly associated with particular aspects of body pigmentation, and the strongest association with aneurysm formation was observed for individual handedness. As for handedness, it is an essential indicator of direction and degree of individual brain lateralization, which in turn is correlated with several aspects of body pigmentation: aberrant and limited migration of cephalic neural crest cells, defective/reduced melanin production, and/or dysfunctional melanocortin receptors, for instance, may be considered as a common basis of two different but related manifestations, that is, reduced pigmentation/albinism and various anomalies in brain lateralization, such as in the motor system manifested as ambidexterity or left-handedness [28–31]. Brain lateralization in turn is clearly associated with several immune functions, as was shown in a series of studies in animals and humans, using various methods (e.g., handedness tests, resections of the cortex, transcranial magnetic stimulation, and EEG analyses) and concerning parameters and processes of both the humoral and cellular immune system (e.g., S-IgA, B and T cells, and natural killer cells) [35–40]. Additionally, a relationship between different levels of specific proinflammatory cytokines (interleukin 1*β* and interleukin 6) in left versus right cortices and motor lateralization has also been demonstrated [41, 42]. Consequently, the strong association between handedness and aneurysm formation observed in the present study may result from different dispositions between left- and right-handers to regulate inflammation in brain arteries, though we cannot, however, explain in detail the immunological processes behind this observation.

One possible explanation relates to an important functional characteristic of melanocytes: recent evidence indicated distinct differences in the antioxidative efficiency of the melanocyte system between individuals. These differences are caused by the melanocortin 1 receptor which is highly polymorphic and a major contributor to the diversity of human pigmentation. Some variants of the MC1R gene are strongly associated with light pigmentation and especially with red hair color in humans (i.e., the RHC alleles), and melanocytes with such MC1R mutations were recently shown to have diminished ability to cope with oxidative stress [43]. Therefore, lighter compared to darker pigmented individuals should be more susceptible to oxidative stress and, consequently, to the formation of intracranial aneurysms. Additionally, there is recent evidence of a general vulnerability of vessels in individuals with MC1R variants. Red- compared to dark-haired volunteers reported significantly more bruising, but without significant differences in a series of coagulation tests, that is, with no abnormalities in hemostasis [44]. Similarly, the usually stronger pigmentation of the anogenital region and areolae compared to the surrounding skin may be attributed to protective functions of these areas. In a forensic sexual assault examination in women after consensual sexual intercourse, for instance, anogenital injury prevalence and frequency varied by the extent of pigmentation; that is, lighter skin color rather than race was a strong predictor for increased external anogenital injuries [45].

Beyond that, the importance of inflammatory processes in intracranial aneurysm formation is further substantiated by the results of recent studies showing that frequent use of acetylsalicylic acid both attenuates inflammation in the walls of human cerebral aneurysms [46] and, thereby, may also reduce the risk of intracranial aneurysm rupture [47]. However, several successful attempts have already been reported targeting the melanocortin receptors themselves as a novel strategy to control inflammation [48]. The therapeutic potential of *α*-MSH and other receptor ligands was demonstrated, for instance, in proteinuria [49], uveitis [50], and skin inflammation [51]. Treatment effects concerning disordered vasculature are, however, the most important for the present study. A synthetic MC1R agonist (BMS-470539), for instance, was demonstrated to inhibit leucocyte trafficking in the inflamed vasculature [52], and the natural agonist *α*-melanocyte-stimulating hormone was shown to regulate vascular NO availability and to protect against endothelial dysfunction [53].

Agonists of the melanocortin system, therefore, may prospectively be seen as efficient therapeutic strategies to fight against developing inflammation in brain arteries and, most importantly, to prevent aneurysm rupture. The logic behind such a strategy, however, requires empirical evidence that particular aspects of body pigmentation, that is, specific processes and functions of the melanocortin system, are significantly associated with the incidence of intracranial aneurysms. Such associations were documented for the first time by the present study which, therefore, adds some new aspects to etiological models of intracranial aneurysm formation.

With respect to the genetic characteristics of the various aspects of body pigmentation, the present results also add some information concerning possible genetic determinants of the observed association: the anti-inflammatory properties of melanocytes arise mainly from the *α*-MSH hormone which is mediated by the MC1R receptor. The MC1R receptor and its genetic mutations, again, are the main source of individual variation in hair and skin pigmentation, a fact well documented in the literature (e.g., [54, 55]). However, in most genetic studies, no association of MC1R with eye color was observed [56–58]. Remarkably, this distinction is also reflected in the results of the present study: significant effects for hair color, but not even a trend in the data concerning eye color. Consequently, the common underlying cause of both lighter pigmentation and an increased susceptibility to aneurysm formation may be seen (among others) in particular polymorphisms of the MC1R gene. Such pleiotropic effects, however, must be demonstrated directly by analyzing the specific genetic variations in patients with intracranial aneurysms in future studies.

Based on the data of Sample II, a rough estimate of the predictive power was finally done with those variables which had proven to be significant in the logistic regression. This analysis resulted in estimations of about 95% and 87% for sensitivity and specificity, respectively. These figures are quite comparable with or even higher than those of other procedures widely used in clinical screening, for instance, faecal occult blood tests for the detection of colorectal cancer in an average-risk screening population (estimated sensitivities ranging from 6% to 83% and specificities ranging from 65% to 99%) [59]. Despite a preliminary and very rough estimation, results indicate that these variables, that is, certain facets of pigmentation and individual handedness, may also have a role as part of clinical screening procedures in detecting intracranial aneurysms in the future.

There are several limitations of the present study. The main sources of imprecision result from the relatively small sample size, especially in Study II. First, this fact specifically concerns facultative and constitutive skin pigmentation. Whereas one of the estimated odds ratios for skin phototype reached significance, logistic regression failed to show an effect for skin color, although a (nonsignificant) trend in the frequency distributions for the three categories of skin color can be observed in the expected direction (distribution in patients opposed to that in controls). However, confidence limits for both skin pigmentation variables are unrealistically high indicating a large portion of error variance caused by the small sample size. This is especially true for the effect of handedness: the highly significant odds ratios for both handedness categories as well as the significant overall trend indicate fundamental importance of this variable. The actual figures, however, appear highly implausible. Future studies determined on a much larger data base will provide more realistic estimates for the common variance between brain lateralization, body pigmentation (both mirroring characteristics and common pathways in the development of the cephalic neural crest), and the formation of intracranial aneurysms. Secondly, a further source of imprecision relates to the quantification of pigmentary traits. With the exception of skin color, pigmentation variables have been based on subjective phenotypes using categorical scales in estimating the level of pigmentation. In future studies, objective and quantitative measures of skin, hair, and eye color should be collected by using, for instance, reflectance or digital spectroscopy and, thereby, excluding a further source of error variance. Finally, our study provides only indirect evidence of an association between the melanocyte system and the formation of aneurysms. As already mentioned above, effects of the melanocyte system on aneurysm formation should be demonstrated directly by analyzing specific genetic variations in patients and controls.

## 7. Conclusions

A significant association between particular aspects of body pigmentation and the incidence of intracranial aneurysms was demonstrated in the present study. Odds ratios increased with a decreased pigmentation of hair and a reduced ability to adjust melanization of epidermal cells after sunlight exposure (“skin photo-type”). Results for hair color were validated in a second independent sample of cases and controls, and the replication study yielded virtually identical results. In addition, individual handedness as a neurophysiological correlate of developmental pigmentation processes was also strongly associated with aneurysm formation; that is, the degree of association increased with an increasing shift from right- to left-handedness.

## Figures and Tables

**Table 1:** Summary of sample characteristics.

	Patients	Controls
	*n* (%)	*n* (%)
Sample I	53	136

Sex		
Males	16 (30.2)	68 (50.0)
Females	37 (69.8)	68 (50.0)
Smoking status		
Smokers	34 (64.2)	80 (58.8)
Nonsmokers	19 (35.8)	56 (41.2)
Age (mean/SD)	55.9 ± 13.2	32.7 ± 8.7

Sample II	58	38

Sex		
Males	17 (29.3)	25 (65.8)
Females	41 (70.7)	13 (34.2)
Smoking status		
Smokers	41 (70.7)	22 (57.9)
Nonsmokers	17 (29.3)	16 (42.1)
Age (mean/SD)	49.9 ± 11.5	69.9 ± 9.7

**Table 2:** Results of logistic regression in Sample I.

Explanatory variables	Patients *n* (%)	Controls *n* (%)	Adjusted odds ratio^†^ (95% CI)	*P* ^#^ (*R* ^2^+)^§^
Sample I	53	136		

Hair color				
Dark brown to brown	12 (22.6)	60 (44.1)	1.0	0.001 (5.60)
Light brown to dark blond	20 (37.8)	64 (47.1)	3.11 (0.86–11.27)	
Blond to strawberry blonde/red	21 (39.6)	12 (8.8)	23.50*** (3.99–138.24)	
Eye color				
Dark color	21 (39.6)	56 (41.2)	1.0	0.40 (0.30)
Mixed color	10 (18.8)	31 (22.8)	1.07 (0.29–3.96)	
Light color	22 (41.6)	49 (36.0)	0.59 (0.18–1.96)	

^†^Odds ratios were adjusted for age, sex, and smoking (*P* values for these covariates were 0.001, 0.040, and 0.114, resp.); ^#^ *P* for trend; ^§^explained variance (%) by the respective variable (Nagelkerke *R* ^2^+); *significance for odds ratios: ***P < 0.001.

**Table 3:** Results of logistic regression in Sample II.

Explanatory variables	Patients *n* (%)	Controls *n* (%)	Adjusted odds ratio^†^ (95% CI)	*P* ^#^ (R^2^+)^§^
Sample II	58	38		

Hair color				
Dark brown to brown	12 (20.7)	25 (65.8)	1.00	0.001 (8.10)
Light brown to dark blond	23 (39.7)	10 (26.3)	2.36 (0.44–12.78)	
Blond to strawberry blonde/red	23 (39.6)	3 (7.9)	29.11*** (3.78–224.32)	
Eye color				
Dark color	14 (24.1)	13 (34.2)	1.00	0.58 (0.09)
Mixed color	17 (29.3)	6 (15.8)	2.06 (0.30–14.19)	
Light color	27 (46.6)	19 (50.0)	1.70 (0.35–8.32)	
Skin color				
Dark	15 (25.9)	17 (44.7)	1.00	0.68 (0.02)
Medium	19 (32.8)	13 (34.2)	1.43 (0.30–6.82)	
Light	24 (41.3)	8 (21.1)	1.43 (0.28–7.41)	
Skin phototype				
Rarely/never burns, tans profusely (V + VI)	6 (10.3)	22 (57.9)	1.00	0.04 (0.48)
Burns moderately/minimally, tans moderately (III + IV)	19 (32.8)	12 (31.6)	2.08 (0.43–10.05)	
Burns easily, tans never/minimally (I + II)	33 (56.9)	4 (10.5)	6.08* (1.11–33.45)	
Handedness				
Pronounced right-handed	8 (14.0)	24 (63.2)	1.00	0.001 (11.40)
Moderate right-handed	20 (35.1)	11 (28.9)	22.36** (2.64–189.32)	
Bimanual or left-handed	29 (50.9)	3 (7.9)	64.09*** (5.40–760.69)	

^†^Odds ratios were adjusted for age, sex, and smoking (*P* values for these covariates were 0.001, 0.009, and 0.558, resp.); ^#^ *P* for trend; ^§^explained variance (%) by the respective variable (Nagelkerke *R* ^2^+); *significance for odds ratios: *P < 0.05, **P < 0.01, and ***P < 0.001.

**Table 4:** Results of logistic regression in data combined from Samples I and II.

Explanatory variables	Patients *n* (%)	Controls *n* (%)	Adjusted odds ratio^†^ (95% CI)	*P* ^#^ (*R* ^2^+)^§^
Samples I + II	111	174		

Hair color				
Dark brown to brown	24 (21.6)	85 (48.9)	1.0	0.001 (13.40)
Light brown to dark blond	43 (38.7)	74 (42.5)	2.84** (1.45–5.58)	
Blond to strawberry blonde/red	44 (39.6)	15 (8.6)	10.82*** (4.71–24.87)	
Eye color				
Dark color	35 (31.5)	69 (39.7)	1.0	0.55 (0.20)
Mixed color	27 (24.3)	37 (21.3)	1.73 (0.85–3.56)	
Light color	49 (44.1)	68 (39.1)	1.22 (0.66–2.25)	

^†^Odds ratios were adjusted for age, sex, and smoking (*P* values for these covariates were 0.001, 0.001, and 0.018, resp.); ^#^ *P* for trend; ^§^explained variance (%) by the respective variable (Nagelkerke *R* ^2^+); *significance for odds ratios: **P < 0.01, ***P < 0.001.
